# Should Steroid Therapy Be Necessarily Needed for Autoimmune Pancreatitis Patients with Lesion Resected due to Misdiagnosed or Suspected Malignancy?

**DOI:** 10.1155/2014/253471

**Published:** 2014-01-22

**Authors:** Wenchuan Wu, Xiuzhong Yao, Chao Lin, Dayong Jin, Dansong Wang, Wenhui Lou, Xinyu Qin

**Affiliations:** ^1^Department of General Surgery, Zhongshan Hospital, Fudan University, Shanghai 200032, China; ^2^Department of Radiology, Zhongshan Hospital, Fudan University, Shanghai 200032, China

## Abstract

To explore whether steroid therapy should be needed for autoimmune pancreatitis patients after operation, eight AIP patients receiving operation were enrolled in this study from January 2007 to July 2013. All patients underwent liver function, CA19-9, and contrast-enhanced CT and/or MRI. Tests of IgG and IgG4 were performed in some patients. Tests of serum TB/DB, **γ**-GT, and **γ**-globulin were undergone during the perioperative period. Six cases receiving resection were pathologically confirmed as AIP patients and two were confirmed by intraoperative biopsy. For seven patients, TB/DB level was transiently elevated 1 day or 4 days after operation but dropped below preoperative levels or to normal levels 7 days after operation, and serum **γ**-GT level presented a downward trend. Serum **γ**-globulin level exhibited a downward trend among six AIP patients after resection, while an upward trend was found in another two AIP patients receiving internal drainage. Steroid therapy was not given to all six AIP patients until two of them showed new lines of evidence of residual or extrapancreatic AIP lesion after operation, while another two cases without resection received steroid medication. Steroid therapy might not be recommended unless there are new lines of evidence of residual extrapancreatic AIP lesions after resection.

## 1. Introduction

Patients with autoimmune pancreatitis (AIP) often receive unnecessary operation due to misdiagnosis for malignances, although they can be cured by steroid therapy without surgery [1, 2]. About 2.5% of patients receiving pancreaticoduodenectomy reported in the literature were pathologically confirmed as AIP [3–5].

Autoantibodies against pancreatic antigen are induced during the pathogenesis of autoimmune pancreatitis (AIP), which causes extensive infiltration of lymphocytes and plasma cells within pancreatic tissue around the pancreatic duct and diffuse enlargement of the pancreas. Similar manifestations may appear in the extrapancreatic organs due to the presence of common antigens. Actually, mechanism for antigens and antibodies of AIP has not been clearly elucidated. The diagnosis and disease severity of AIP are mainly dependent on a comprehensive assessment with serology, morphology, imaging, and therapeutics [6]. Similar to IgG, IgG4 is only the representative of the responsive level of antigen-specific antibody in the serum, although it is a key indicator among the diagnostic criteria for AIP [7, 8]. IgG4 is also related to the degree of AIP but does not directly represent the extent of lymphoplasmacytic infiltration into the pancreas or extrapancreatic organs.

Accordingly, the continuous treatment plans for AIP patients with unnecessary operation, especially for those receiving excision or exclusion, might be based on not only the serum level of IgG4, IgG, or Gamma globulins indicating the level of antibody in the serum considered but also operational styles or the presence of extrapancreatic organs involved. In the current study, we aimed to explore whether the steroid therapeutic should be needed for those autoimmune pancreatitis patients, who received operation due to misdiagnosed or suspected malignancy.

## 2. Patients and Methods

### 2.1. Included Patients

From January 2007 to July 2013, sixteen patients with AIP were treated in our pancreatic surgery center. Among those 16 cases, 8 AIP patients received operation. Six cases received pancreaticoduodenectomy (patients No. 1 to 3, 5, and 8) or distal pancreatectomy (patient No. 4) due to being preoperatively misdiagnosed with malignant tumors. Two cases (patients Nos. 6 to 7) received laparotomy because of failing to exclude malignant tumors, and they finally avoided operation due to definite diagnosis of AIP by intraoperative biopsy. The eight AIP patients above were enrolled in this study. The protocol was approved by the Ethics Committee of Zhongshan Hospital, Fudan University, which conforms to the provisions of the World Medical Association's Declaration of Helsinki in 1995 (as revised in Tokyo 2004). And written informed consents for participation in the study and for the publication of individual clinical details and accompanying clinical images were obtained from all patients.

### 2.2. Biochemical Test and Diagnostic Evaluation

The clinical data of 8 patients was summarized in Table 1. All these eight patients underwent routine examinations, including liver function, CA19-9, and contrast-enhanced CT and/or MRI before operation. Test of IgG was performed in 5 patients (patients Nos. 1 to 2 and 4 to 6), while test of IgG4 was performed in two patients (patients Nos. 4 and 5) (Table 2).

AIP was confirmed by pathological assessment of resection specimens or intraoperative frozen biopsy, meeting the first *Diagnostic Criteria for Autoimmune Pancreatitis* from Mayo Clinic Center [7, 8], which is lymphoplasmacytic sclerosing pancreatitis or a large number of IgG4-positive cells (≥10 cells/high power field).

The patients with preoperative suspected AIP in this study were defined as “probable AIP,” which were only based on clinical data, except serum IgG4. Tests of serum TB/DB, *γ*-GT, and *γ*-globulin were performed during the perioperative period.

### 2.3. Steroid Therapy

For those patients with AIP lesion removed due to preoperative misdiagnosis as malignant tumors, steroid therapy was not recommended unless there were new lines of evidence of residual AIP lesion or extrapancreatic lesion; steroid medication was given to those AIP patients confirmed by intraoperative frozen biopsy, because their AIP lesions were not removed.

Initial dose of prednisone was 40 mg/day for two–four weeks, followed by a reduction of 5 mg each week. Every four weeks, CT scan or MRI examination was performed to evaluate the responses of pancreatic mass and narrowing of main pancreatic duct.

## 3. Results

### 3.1. Clinic Characteristics

All eight AIP patients were male. Their median age was 58 years (49–76 years). Initial symptoms were jaundice (3 patients), epigastric discomfort (1 patient), and abdominal pain (2 patients), respectively, and two patients had no compliment due to diagnosis by routine checkup. No allergy history to any foods and drugs was recorded, except one (patient No. 3) with penicillin allergy. All patients denied history of diabetes, Sjogren's syndrome (except patient No. 5), primary sclerosing cholangitis, ulcerative colitis, systemic lupus erythematosus, Crohn's disease, and other autoimmune diseases (Table 1).

### 3.2. Biochemical Test

CA19-9 was normal in 5 patients or slightly elevated in 3 patients, with only one case (patient No. 2) more than 100 U/mL. Serum *γ*-globulin level increased in 7 of 8 AIP patients. Patient No. 5 might belong to a subtype of AIP that often exhibits normal serum levels of IgG4 and *γ*-globulin. From the limited data of five cases, we found that serum level of IgG was elevated in AIP patients (except Patient No. 5). Patient No. 4 displayed elevated serum IgG4 level 7 months after operation.

### 3.3. Diagnostic Evaluation

According to the first Diagnostic Criteria for Autoimmune Pancreatitis from Mayo Clinic center [7, 8], eight patients were pathologically confirmed as AIP patients. Among them, 6 cases were approved by resection specimens and 2 by intraoperative frozen biopsy. Resection specimens of 6 AIP patients demonstrated a diffusely enlarged and firm pancreas with marked interstitial fibrosis. The cut face of pancreas appeared pale yellow, and segmental narrowing of the main pancreatic duct was found with upstream dilatation, as well as stenosis of the lower common bile duct with upstream bile duct dilatation. No significant mass was observed except enlarged pancreatic acinar. Microscopic appearance of AIP displayed a dense lymphoplasmacytic infiltration of the pancreatic parenchyma surrounding the common bile duct and main pancreatic duct with secondary fibrosis and reduced pancreatic acini. Intraoperative frozen biopsy disclosed chronic pancreatitis with dense lymphoplasmacytic infiltration and fibrosis. Immunohistochemistry exam showed that there were a large number of IgG (IgG4-) positive lymphocytes.

A heterogeneous and ill-defined mass within the pancreas was disclosed on the contrast-enhanced CT or MRI images, with partial involvement of the blood vessels around the pancreas. MRCP demonstrated segmental narrowing and dilatation of main pancreatic duct.

### 3.4. Biochemical Changes in the Perioperative Period

Six cases with AIP received PD or DP. Two cases received the internal drainage operation for obstructive jaundice after AIP was confirmed by intraoperative frozen biopsy. Changes of serum TB/DB and *γ*-GT levels in one patient (patient No. 4) were found within normal ranges during the perioperative period because CBD was not involved in the focal AIP lesion in the pancreatic body and tail. For another 7 cases with AIP mainly in the head of pancreas, whether PD or the internal drainage was performed, TB/DB level was transiently elevated 1 day or 4 days after operation but dropped below preoperative levels or to normal levels 7 days after operation. Serum *γ*-GT level presented a downward trend, which was similar to those patients with pancreatic head cancer (data not showed). Serum *γ*-globulin level exhibited a downward trend in six AIP patients after resection. However, for the other two AIP patients who received the internal drainage, their postoperative serum *γ*-globulin kept on higher level and displayed an upward trend (Table 3).

### 3.5. Steroid Therapy

Six AIP patients received pancreaticoduodenectomy (patients Nos. 1 to 3, 5, and 8) or distal pancreatectomy (patient No. 4) for being preoperatively misdiagnosed with pancreatic or biliary malignancies. Steroid therapy was not given to these patients after operation due to removal of the main lesion. However, 2 months after operation, one case (patient No. 1) appeared to have obstructive jaundice, mildly elevated *γ*-GT, and significant increase in *γ*-globulin, IgE and IgG, which raised the possibility of coexisting sclerosing cholangitis, and then was treated with prednisone with a dose of 40 mg/day. Two weeks later, jaundice disappeared and the level of *γ*-globulin decreased; then the dose reduced at 5 mg each week. And 7 months after distal pancreatectomy, another case (patient No. 4) displayed elevated serum IgG4 level and received prednisone therapy, for being considered about other AIP lesions existed in the residual pancreas.

**Figure 1:**
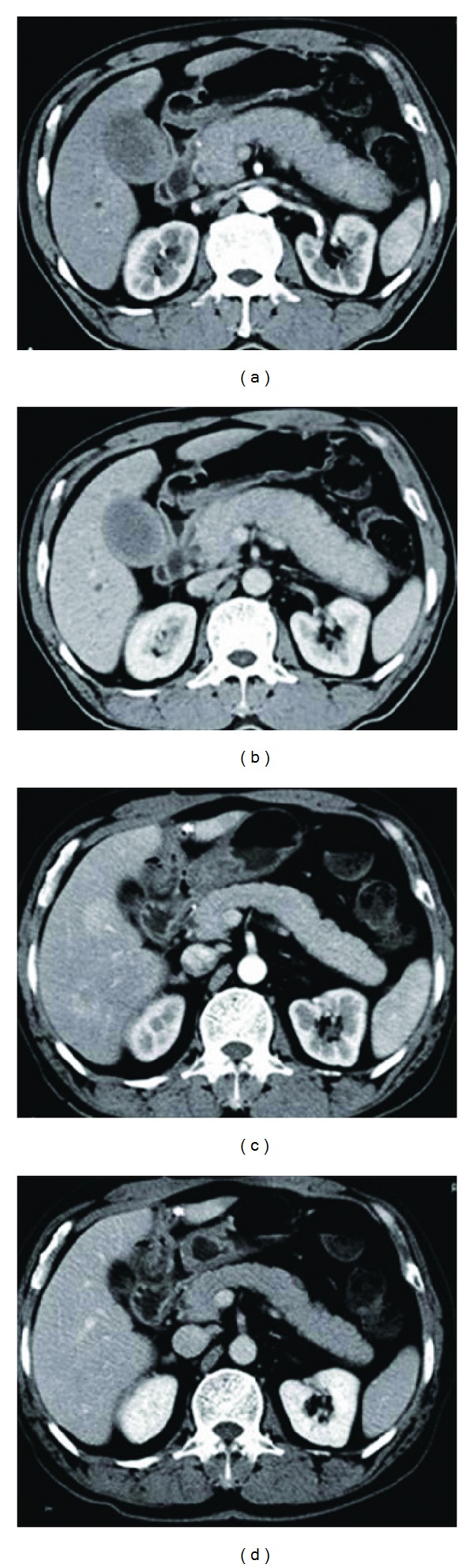
Imaging changes before and after steroid therapy in patient No. 6. Pretreatment: the arterial phase (a) and venous phase (b) of contrast-enhanced computed tomography both revealed a diffusely swollen pancreas with a “capsule-like rim” and “sausage-like” appearance. After internal drainage and a 3-month steroid therapy, the arterial phase (c) and venous phase (d) of contrast-enhanced showed that the swollen status of pancreas had been obviously reduced.

Two preoperative suspected AIP patients were confirmed by intraoperative frozen biopsy (patients Nos. 6 and 7) and received the internal drainage for obstructive jaundice, which successfully avoided the unnecessary resection. Steroid medication was given to them because the AIP lesions were not removed. Initial dose of prednisone was 40 mg/day for four weeks, and then followed by a reduction of 5 mg each week. For patient No. 6, the diffusely swollen pancreas with “capsule-like rim” and “sausage-like” appearances had not been obviously observed after internal drainage, followed by 3 months of steroid therapy (Figure 1).

## 4. Discussion

Steroid treatment is the mainstay of management of AIP, with characteristic dramatic response [1]. But exact indications and optimal regimens of steroid therapy for AIP are still not well established. Recently, a consensus proposal for the treatment of AIP was recommended by Japanese researchers [9], which suggested that steroid therapy started with 30–40 mg (0.6 mg/kg body weight) of oral prednisolone per day for 2–4 weeks, and then the dose would be tapered to a maintenance dose (5.0–7.5 mg prednisolone per day) for a total of 2-3 months [10, 11]. However, this proposal does not include the treatment of a group of AIP patients with the main lesions resected. AIP patients are often misdiagnosed with pancreatic or biliary malignancies [2] and received the unnecessary resection [3], since this pathological entity demonstrates nearly the same clinical manifestations as pancreatic or periampullary cancer does. Accordingly, it needed to be addressed clearly whether steroid therapy should be recommended to those patients with AIP lesions removed? However, the published literature on this question is limited.

In principle, the treatment modality of AIP can be classified into medical and surgical therapy. Resective surgery is usually done without preoperative suspicion for AIP, and its role is not yet clarified. It might be curative by removing the lesion completely in the mass-forming AIP [12]. Now, it is generally agreed that steroids should be offered to AIP patients with “active” disease [13]. And evaluation of the AIP disease activity requires biochemical blood tests such as serum *γ*-globulin, IgG and IgG4, imaging findings, and clinical manifestations such as jaundice and abdominal discomfort [10]. According to this evaluation standard, we thought that AIP patients receiving resection in this study were cured and did not need steroid therapy any more. In our data, the clinical symptom, such as jaundice and abdominal discomfort, was eliminated after PD or DP because the main AIP lesions were resected and no lesions were observed on imaging examination. Meanwhile, biochemical tests during the perioperative period show a downward trend of p serum *γ*-globulin. Therefore, it is reasonable to deduce that the disease activity of AIP patients with lesion resected had been totally controlled and turned into “inactive” status, though we cannot exclude the possibility of involvement of the residual pancreas or *extrapancreatic* organ after surgery [14, 15]. Accordingly, for those patients with AIP lesions removed, steroid therapy was unnecessary.

For the other two AIP patients confirmed by intraoperative biopsy, the clinical symptoms such as jaundice and abdominal discomfort could be eliminated by internal drainage. But according to the same evaluation standard, the AIP disease activity kept on a high level, not lower than that before operation, mainly because the AIP lesion had not been resected. As a result, the perioperative serum *γ*-globulin disclosed an upward trend. Therefore, steroid therapy was recommended for those patients without AIP lesions resected.

Up to now, the duration of maintenance therapy has still been controversial. Chari [11] recommended a total of 12 weeks of steroid therapy. Although there was a 30–40% relapse rate following withdrawal of steroids, the second course of steroids was given to such patients when relapse occurred [16]. But there is also another suggestion about the maintenance therapy. AIP patients often show relapse of pancreatic and/or extrapancreatic manifestations within 3 years [17, 18] from the initiation of the steroid therapy; thus, the steroid therapy was recommended to be maintained for at least 3 years [19]. And when AIP patients relapse during or after steroid therapy, steroid therapy should be restarted with a higher dose of prednisolone [20].

We agree on the 12 weeks of steroid therapeutic strategy for AIP. Long time steroid therapy might cause a lot of side effects. And on the other hand, it is still unclear if long term steroid therapy could alter the natural history of AIP or prevent relapse in the pancreas [11]. So, the period of maintenance steroid therapy should not be too long. Based on this point, we develop our therapeutic strategy for AIP patients receiving resection; no dose of prednisolone was recommended. However, relapse of pancreatic and/or extrapancreatic manifestations should be considered and prednisolone should be recommended if the patients appeared to have elevated serum level of *γ*-globulin, IgG, IgG4, or clinical manifestations such as jaundice and abdominal discomfort after operation.

According to the study by Weber et al. [21], relapse rate after pancreatectomy was 28%. In our series, patients Nos. 1 and 4 were suspected for extrapancreatic sclerosing cholangitis and residual AIP lesions at the time of 2 and 7 months after operation, respectively. Thus, a standard prednisone therapy was given to those two patients just like relapsed AIP patients.

In one word, we agree on the steroid therapeutic strategy for AIP, less than 3-month periods of maintenance therapy. Specially, we imply that steroid therapy may not be recommended unless there are new lines of evidence of residual AIP lesion or extrapancreatic lesion after operation.

## Figures and Tables

**Table 1:** Clinic characteristics of AIP patients.

Case	Sex	Age (yr)	Initial diagnosis	Diagnostic criteria	Initial symptoms	Operation	Steroid therapy	Follow-up months
1	Male	67	Pancreatic head cancer	^*①*†^	Jaundice	PD	Yes*	80
2	Male	51	Lower common bile duct cancer	^*①*†^	Jaundice	PD	No	60
3	Male	76	Pancreatic head cancer	^*①*†^	Routine checkup	PD	No	55
4	Male	58	Pancreatic body cancer	^*①*†^	Epigastric discomfort	DP	Yes^§^	21
5	Male	58	Pancreatic head cancer	^*①*†^	Abdominal pain	PD	No	32
6	Male	64	Suspected AIP	^*①*‡^	Jaundice	Biopsy, ID	Yes	50
7	Male	49	Suspected AIP	^*①*‡^	Abdominal pain	Biopsy, ID	Yes	57
8	Male	54	Pancreatic head cancer	^*①*†^	Routine checkup	PD	No	33

Note: PD: pancreaticoduodenectomy; DP: distal pancreatectomy; ID: internal drainage; ^*①*^meaning the patient meets the first Diagnostic Criteria for Autoimmune Pancreatitis from Mayo Clinic Center, that is, lymphoplasmacytic sclerosing pancreatitis or a large number of IgG4-positive cells (≥10 cells/high power field), depending on pathology assessment of resection specimens (^†^) or intraoperative frozen biopsy (^‡^); *60 days after operation; ^§^7 months after operation.

**Table 2:** Serological tests of AIP patients.

Case	TB (*μ*mol/L) (3.4~20.4)	DB (*μ*mol/L) (0~6.8)	*γ*-GT (U/L) (11~50)	Gamma globulin (%) (11.8~18.8)	CA19-9 (U/mL) (<37)	IgG (g/L) (7~16)	IgG4 (g/L) (0.03~2)
1	147.5	87.4	1404	23.4	97.2	NA	NA
2	21.2	12.2	726	19.7	123.4	NA	NA
3	31.9	22.4	624	21.3	26.2	NA	NA
4	5.1	1.5	46	25.0	22.9	NA	NA
5	10.6	4.5	101	16.4	20.0	10.7	0.86
6	266.9	219.9	86	25.8	22.7	26.4	NA
7	6.0	3.0	88	20.6	30.4	NA	NA
8	4.5	1.7	47	22.5	53.9	NA	NA

Note: TB: total bilirubin. DB: direct bilirubin. *γ*-GT: *γ*-glutamyltransferase. NA: not available.

**Table 3:** Biochemical changes for AIP patients receiving operation in the perioperative period.

Case	TB/DB (*μ*mol/L)			*γ*-GT (U/L)			*γ*-globulin (%)			IgG (g/L) (7~16)	IgG4 (g/L) (0.03~2)
	Day 1	Day 4	Day 7	Day 1	Day 4	Day 7	Day 1	Day 4	Day 7		
1	207.4/115.3	294.6/155.0	129.6/76.3	905	478	379	21.9	20.5	19.1	28.1^♂^	NA
2	17.3/10.0	11.9/8.9	10.8/7.7	344	251	186	19.9	18.6	17.9	18.8^‡^	NA
3	20.5/12.1	13.9/8.3	9.9/7.4	419	27.9	231	19.7	19.3	18.40	NA	NA
4	10.8/4.5	9.5/4.3	NA^†^	38	29	NA^†^	24.2	23.2	NA^†^	18.9^‡^	13.4^§^
5	25.5/11.0	22.2/14.4	NA^†^	66	71	NA^†^	15.6	12.5	NA^†^	NA	NA
6	224.3/194.3	178.0/155.3	NA^†^	27	28	NA^†^	26.8	27.3	NA^†^	NA	NA
7	22.8/18.7	16.5/14.2	10.4/8.2	80	128	197	23.6	25.4	27.5	NA	NA
8	NA	16/10.3	9.8/5.8	NA	48	44	NA	13	1	NA	NA

Note: TB: total bilirubin; DB: direct bilirubin; *γ*-GT: *γ*-glutamyltransferase; day 1 (4, 7), 1 (4, 7) day(s) after operation; NA: not available; ^†^discharged; ^♂^60 days after operation; ^‡^10 days after operation; ^§^7 months after operation.

## References

[1] Sah RP, Chari ST (2012). Autoimmune pancreatitis: an update on classification, diagnosis, natural history and management. *Current Gastroenterology Reports*.

[2] Agrawal S, Daruwala C, Khurana J (2012). Distinguishing autoimmune pancreatitis from pancreaticobiliary cancers: current strategy. *Annals of Surgery*.

[3] Hardacre JM, Iacobuzio-Donahue CA, Sohn TA (2003). Results of pancreaticoduodenectomy for lymphoplasmacytic sclerosing pancreatitis. *Annals of Surgery*.

[4] Yadav D, Notahara K, Smyrk TC (2003). Idiopathic tumefactive chronic pancreatitis: clinical profile, histology, and natural history after resection. *Clinical Gastroenterology and Hepatology*.

[5] Abraham SC, Wilentz RE, Yeo CJ (2003). Pancreaticoduodenectomy (Whipple resections) in patients without malignancy: are they all ’chronic pancreatitis’?. *American Journal of Surgical Pathology*.

[6] Wakabayashi T, Kawaura Y, Satomura Y (2002). Clinical study of chronic pancreatitis with focal irregular narrowing of the main pancreatic duct and mass formation: comparison with chronic pancreatitis showing diffuse irregular narrowing of the main pancreatic duct. *Pancreas*.

[7] Chari ST, Smyrk TC, Levy MJ (2006). Diagnosis of autoimmune pancreatitis: the Mayo Clinic experience. *Clinical Gastroenterology and Hepatology*.

[8] Chari ST (2007). Diagnosis of autoimmune pancreatitis using its five cardinal features: introducing the Mayo Clinic’s HISORt criteria. *Journal of Gastroenterology*.

[9] Kamisawa T, Okazaki K, Kawa S, Shimosegawa T, Tanaka M (2010). Japanese consensus guidelines for management of autoimmune pancreatitis: III. Treatment and prognosis of AIP. *Journal of Gastroenterology*.

[10] Ito T, Nishimori I, Inoue N (2007). Treatment for autoimmune pancreatitis: consensus on the treatment for patients with autoimmune pancreatitis in Japan. *Journal of Gastroenterology*.

[11] Chari ST (2007). Current concepts in the treatment of autoimmune pancreatitis. *Journal of the Pancreas*.

[12] Frulloni L, Cavallini G (2005). Treatment of autoimmune pancreatitis: the search for an optimal management continues. *Pancreatology*.

[13] Ghazale A, Chari ST (2007). Optimising corticosteroid treatment for autoimmune pancreatitis. *Gut*.

[14] Taniguchi T, Tanio H, Seko S (2003). Autoimmune pancreatitis detected as a mass in the head of the pancreas without hypergammaglobulinemia, which relapsed after surgery: case report and review of the literature. *Digestive Diseases and Sciences*.

[15] Kamisawa T, Egawa N, Nakajima H, Tsuruta K, Okamoto A (2005). Extrapancreatic lesions in autoimmune pancreatitis. *Journal of Clinical Gastroenterology*.

[16] Kamisawa T, Yoshiike M, Egawa N, Nakajima H, Tsuruta K, Okamoto A (2005). Treating patients with autoimmune pancreatitis: results from a long-term follow-up study. *Pancreatology*.

[17] Wakabayashi T, Kawaura Y, Satomura Y, Watanabe H, Motoo Y, Sawabu N (2005). Long-term prognosis of duct-narrowing chronic pancreatitis: strategy for steroid treatment. *Pancreas*.

[18] Kamisawa T, Okamoto A, Wakabayashi T, Watanabe H, Sawabu N (2008). Appropriate steroid therapy for autoimmune pancreatitis based on long-term outcome. *Scandinavian Journal of Gastroenterology*.

[19] Nishimori I, Otsuki M (2009). Autoimmune pancreatitis and IgG4-associated sclerosing cholangitis. *Best Practice & Research: Clinical Gastroenterology*.

[20] Kamisawa T, Okamoto A (2007). Prognosis of autoimmune pancreatitis. *Journal of Gastroenterology*.

[21] Weber SM, Cubukcu-Dimopulo O, Palesty JA (2003). Lymphoplasmacytic sclerosing pancreatitis: inflammatory mimic of pancreatic carcinoma. *Journal of Gastrointestinal Surgery*.

